# Thomas Keith and Ovariotomy

**Published:** 1881-03

**Authors:** J. Marion Sims

**Affiliations:** New York


THOMAS KEITH AND OVARIOTOMY. BY J. MARION SIMS, M.D., LL.D., of New York. From the *American Journal of Obstetrics*, April, 1880.

Dr. Keith, of Edinburgh, did his first ovariotomy in September, 1862. Since then he has operated three hundred and three times. When he published his first series of fifty cases, his remarkable success was supposed to be accidental; and when he published his second series of fifty cases, making a hundred in all, his success so far outstripped that of all other operators that it became a wonder and admiration of surgeons all over the world. Since that time I have felt the greatest curiosity to see him operate. I wished to see in what his method differed from that of all other great ovariotomists. I wished to see if there was anything peculiar to himself to account for his great success. Accordingly, while in London, in the summer of 1874, I wrote to him to ask the privilege of witnessing some of his operations; but unfortunately he had none when I could have gone to Edinburgh. At this time most surgeons were using the clamp to secure the pedicle, but Thomas Keith was using the actual cautery, and it was supposed by many, even by himself, that the cautery was the principal cause of his success.

In 1876 and 1878 I again failed in my endeavor to witness operations by him. In 1879 I wrote to him that I had already written the chapter on ovariotomy for my forthcoming book, but would never consider it complete till I had seen him operate. He accordingly arranged for me to visit Edinburgh on the 1st of July,

1879, and I propose now to give an account of my observations made at that time.

Keith began the use of Lister's antiseptic method in March, 1877. Previously to that, his success had been from eighty-six to ninety per cent.; while that of other operators had gradually crept from sixty-six up to seventy and seventy-five per cent., and in one or two instances to eighty. But under the antiseptic method Keith has cured of his last hundred cases ninety-seven per cent.; seventy-three of these in succession without a single death.

If Keith cured from eighty-six to ninety per cent. before antisepsis, while others were curing seventy or even eighty; and if he now cures ninety-seven per cent. with it, while others cannot get over eighty-eight or ninety with it, then I thought there must be something else beside antisepsis to account for this difference. With this feeling I went to Edinburgh, and I think I have learned the secret of his great success—a secret that he is hardly aware of himself, because he has rarely seen the operation performed by any one else.

Keith is systematic in everything he does. He uses a Lister's spray apparatus with three jets, which works six hours if necessary, and is placed to the left of the patient's head, at a distance of eight or nine feet from the seat of operation. Most other surgeons place it at the feet and to the left. By Keith's plan the spray interferes less with the assistants, and is not expended on their arms and elbows. After operations his sponges are thoroughly washed, and then soaked for ten or twelve hours in a solution of washing soda, which cleanses them of blood and fibrin. Previously to operation, they are soaked in carbolized water (one to twenty). Just before operation they are wrung out of a hot carbolized solution, and put in a tight covered tin pail, and placed near the fire to be kept warm till they are used. His operating table is twenty-two inches wide and thirty-three inches high. In his early operations he used chloroform as an anæsthetic. But in later years he has used ether, and thinks it safer than chloroform, and that recoveries under ether would always be greater than under chloroform, other things being equal. He thinks the depressing effects of chloroform contributed directly to the death of some of his early cases. He carries out Listerism very carefully with hands, instruments and sponges, all well carbolized. He operates usually about eleven o'clock in the day, and the patient is only allowed a little tea and toast at eight in the morning. Ordinarily, he does not put his patients under any long preparatory treatment for the operation. I saw him perform two ovariotomies, and each case came to his infirmary on the day preceding operation. Just before operation he had visited two bad cases of diphtheria. Spencer Wells requires all spectators at his operations at the Samaritan Hospital to sign a pledge saying they had not attended any contagious or infectious disease, or been engaged in dissections or post-mortem examinations for a week. Keith believes that antiseptics protect his patients against all danger of infection, and does not exact from spectators any such pledge. The gentleman who gives ether for him is engaged in a large obstetric practice, and Keith never inquires whether he has puerperal fever or septic cases in hand. For myself, however strongly I believe in the protective power of Listerism, I would certainly prefer to have assistants and spectators clear of all suspicion of contagion.

The idea has gone abroad that Keith is a slow operator, simply because his operations are prolonged beyond the time that would be taken by most surgeons to do like operations. But this is a great mistake, for I have never seen any one cut down to the peritoneal cavity more quickly, though always cautiously, remove a tumor with greater celerity, or close up the external wound more rapidly than Dr. Keith. The time that he dallies is when he comes to arrest hæmorrhage, by ligating bleeding points and clearing out the peritoneal cavity, and when the operation is finished, you involuntarily ask yourself, Could it possibly have been done better? And the answer comes spontaneously: "Impossible."

Keith never hurries; he does nothing for display; he leaves no bleeding points; never closes the wound till he is sure that all oozing has ceased; till he is sure that the peritoneum is perfectly dry. When he performed his first operation in 1862, he was surrounded by old men in the profession, who had the dread of wounding the peritoneum continually before their eyes. He was obliged to break up extensive adhesions, and as a consequence there was a free exudation of blood. Before closing the external wound, he began to sponge out the peritoneal cavity, and suddenly thrust a large sponge down in the pelvis and brought it up saturated with blood. Squeezing it dry, he was about to repeat this process, when they all united in begging him not to do it! From their standpoint of view, there would be more danger of irritating the delicate peritoneum with the sponge than by leaving the blood there to be absorbed. He yielded against his judgment to entreaties, and closed the wound, leaving a large quantity of blood in the peritoneal cavity. On the third day his patient was profoundly septicemic, and in imminent danger. He recognized the source of danger, and had the courage to open the lower angle of the wound by removing two or three sutures. There was an immediate discharge of fetid bloody serum in large quantities, and from that moment the patient began to improve and soon got well. This made a profound impression upon Keith's mind, and he determined from that time never again to leave extravasated blood in the peritoneal cavity if he could possibly remove it. It was not long before he had an opportunity of putting this principle to the test of experiment, for his second case was a very bad one with extensive adhesions. He had to tie many vessels and bleeding points. There was a large exudation of blood in the pelvic cavity, and he sponged it all out thoroughly, after which he closed up the external wound, and his patient recovered without a single bad symptom. From this time he adopted the principle of never closing the external wound till he had controlled all oozing of blood and made sure that the peritoneal cavity was dry and clean.

To the adoption of this principle at the very commencement of his work, more than to any other one thing, was certainly due the great success he achieved before he began the antiseptic method; and its conjunction with antiseptics is the cause of his unparalleled success under Listerism. The first case I saw him operate on was a very unfavorable one. The patient was old and feeble, the tumor multilocular, and universally adherent. With a few strokes of the knife, the peritoneum was reached. He used no scissors, no tenaculum, no dissecting forceps, no director. But three or four hemostatic forceps were hanging to the edges of the incision after it was completed. The tumor was so intimately adherent to the abdominal parietes that it was difficult to define the line of union. I have never seen anything more unyielding; it was almost like cartilage; it could be separated only by dissecting with a knife; it was, therefore, necessary to open the tumor and tear out its multiple contents with the hand before the sac could be separated from the adjacent parts.

His trocar tube is a half inch in diameter, and he used Nélaton's forceps to clasp the sac-walls. The pedicle was long and narrow. He clasped it with locked forceps, and cut away the sac before he separated remaining adhesions. The omentum was firmly adherent to the tumor by the whole of its free border, and, as it was separated, it was necessary to tie it in segments, some as large as the finger, with twelve separate catgut ligatures. After the omentum was disposed of, he broke up the remaining adhesions to the parietes on each side and to the intestine and meso-colon, applying catgut ligatures whenever it was necessary to restrain hæmorrhage.

When he had ligated all bleeding points, he turned his attention to the pedicle. It was long and slender, and he would have transfixed it with a soft iron wire and tied on each side if I had not been present; but to gratify me he took it off with the actual cautery. He uses carbolized catgut ligatures to all bleeding points in the peritoneal cavity, except for the pedicle, and for this he has at different times used the clamp, the ligature, and the cautery; but he prefers the cautery. In this instance, he encircled the pedicle with a Baker-Brown cautery clamp, which he screwed up with a moderate degree of firmness. He had a half-dozen cautery irons ready heated in a little portable charcoal furnace. Taking one of the irons out of the furnace, he found it at a red-heat, which was too hot, and he dipped it in cold water till it was as dark as if it had not been heated at all. He then placed its hatchet edge against the clamped pedicle, and by gentle motion back and forth he burned it off close to the upper surface. Its burning made a creaking, whistling noise. He then took another hot iron and dipped it in cold water till it was of a brown heat, and polished the burnt edge of the pedicle till it was quite smooth and even with the surface of the clamp. He then cooled off the clamp with a sponge in cold water. Afterward he caught the pedicle with Koeberle's hemostatic forceps underneath the clamp, then unscrewed the clamp and separated its blades gently and very slowly. He watched the pedicle for a moment to see if there was any bleeding, and, as there was none, he removed the clamp entirely. The portion of the pedicle which had been compressed by the clamp had been squeezed so forcibly for ten or fifteen minutes that it looked transparent, like a bit of clarified horn. The part embraced by the clamp was about an inch and a half long, one-fourth of an inch wide, and about one-sixteenth of an inch thick. The burnt edge was browned, but had no thickened, charred substance sticking to it as I expected. I suggested to Keith that the forcible compression of the clamp might have been a sufficient hemostatic in a case like this, without the cautery. He said he had tried it, and it could not be depended upon, and that the cautery was essential to amalgamate the edges of the clamped pedicle. The pedicle was temporarily held in the lower angle of the wound by his hemostatic forceps, one on each side, just below the border that had been compressed by the clamp, while he proceeded (as the German surgeons say) to "make the toilet of the peritoneal cavity." He cleared out the peritoneal cavity with sponges, removing several ounces of extravasated blood and serum. Then he began to hunt bleeding points on surfaces from which adhesions had been broken up. He tied one point and then another, and another which had given rise to the smallest possible transudation of blood. Then the whole pelvic cavity was again sponged out, and again he searched for oozing vessels until he tied perhaps twenty points. He continued the search and ligation of seemingly unimportant little oozing points long after any other surgeon would have hastily closed up the external wound, leaving something to chance. Not so with Keith; he explores and re-explores, and you wonder why he does not at once finish the operation, when suddenly he seizes a point moistened with fresh blood, and throws a ligature around it. And so he goes on, till he feels sure that there is not a point left unsecured from which bleeding, after the establishment of reaction, could possibly take place.

At last he is ready to close the external wound. He places, according to the plan of Spencer Wells, a broad, flat sponge, perhaps six inches long by three or four wide, within the peritoneal cavity overlying the exposed intestines. The object of this is to protect the intestines against cold during the time of passing the sutures, and also to protect them from any blood that may drop into the peritoneal cavity from the needle punctures. He then passes two sutures at the upper angle of the wound, and two at the lower, according to the method of Spencer Wells; each suture having a needle at either side, and each needle passed from within the peritoneal membrane, and out through the skin. The intervening sutures were passed rapidly with an awl-shaped needle, six inches long, with an eye at the point, according to the method of Peaslee. The sutures were passed through the entire thickness of the abdominal walls, not more than from a quarter to the third of an inch from the edge of the incision, and embracing the peritoneal membrane. When the sutures were all introduced, the upper half of them were drawn up in their middle portions into the upper angle of the incision, and the lower angle, where they were held by assistants, thus making clear the opening into the peritoneal cavity by which he removed the sponge that had been placed there for protection, before the introduction of the sutures. When the sponge is removed, if it is bloody he immediately begins the search for bleeding points; but if it is dry the wound is ready for closure. But before doing this he hastily thrusts a small sponge, held by a locked forceps, to the bottom of the pelvic cavity, to determine if it is still dry. If all is well, the sutures are all drawn tight, and each tied separately, while an assistant presses the relaxed abdominal walls together with his hands, in a line under and parallel with the course of the wound. The sutures are then cut off, each within an inch or two of the knot. In this case, seven deep carbolized silk sutures were passed, with five intermediate surface sutures of horse's hair. The wound was dressed with thymol gauze, covered with cotton-wool and a flannel band. In cases like this, he formerly used a glass drainage-tube before the days of antisepticism, but now he does it only occasionally. The operation was begun at twenty-five minutes after eleven, and finished at twenty-eight minutes after twelve—one hour and three minutes.

It is often the habit with novices in ovariotomy to saturate their patients with morphine or some preparation of opium for the first few days after operation; but Keith and Spencer Wells long ago learned that this practice was not only useless, but injurious, and their custom is to order twenty drops of laudanum, or its equivalent, to be given by the rectum after the patient recovers from the anæsthetic if there be pain enough to require it, to be repeated if necessary. For the first forty-eight hours the patient takes no nourishment. He gives only brandy and ice as they may be necessary. At the end of this time he allows light nourishment, such as beef-tea and milk, and in a day or two the bowels may be moved by enema, unless there is something to contraindicate its use. If all goes on well, a more nutritious diet may now be allowed. Keith attaches much importance to a free discharge of flatus from the rectum, and always watches this symptom with great anxiety.

I saw Keith perform his second operation on the 4th of July, 1879. (Here follow copious notes of each step of Dr. Keith's operation, made by Dr. Sims on the spot, which we need not here insert.)

The tumor weighed fifty-six pounds, thirty-four fluid, twenty-two solid. The india rubber sheeting inclosing the sponges at the mouth of the drainage-tube is unfolded from time to time; the sponges removed and squeezed over a graduated measure, and the quantity of fluid noted. This must be done more or less frequently, according to the amount of bloody serum discharged. At first it may be necessary to do this every four or five hours. As the quantity of fluid discharged decreases, this may be done at longer intervals. Keith never finds it necessary to make intraperitoneal injections, as is so frequently practiced here; it seems that the fluid finds it way out through the drainage tube as rapidly as it is extravasated.

We are indebted to Koeberle for drainage by abdominal incision. He first used it in 1867. But he has now, I believe, given it up almost entirely. Keith learned its use from Koeberle in 1868. He used it more frequently before he began antiseptics than he does now; but he says there are exceptional cases in which he thinks drainage is absolutely essential to the safety of the patient. Spencer Wells and Thornton, since they adopted antiseptics, do not resort to the drainage-tube; they exclude it on the principle that Listerism renders all intra-peritoneal effusions, whether blood or serum, aseptic. Being aseptic, they can be absorbed in immense quantities, without danger to the life of the patient. But suppose we have not succeeded in rendering the peritoneal effusions perfectly aseptic, then we know that, in very small quantities, their absorption may be attended with great danger. How shall we then determine whether it will be necessary to use the drainage-tube or not? Are there any indications to guide our judgment in this matter?

In the two cases upon which I saw Keith operate, each had extensive adhesions to be broken up. In the first, he did not use the drainage-tube; in the second, he did. After leaving Edinburgh, I wrote to ask him the explanation of this difference of practice in two cases that were so much alike, and to ask if he could lay down a principle of action to guide us under all circumstances. In his reply, dated Edinburgh, July 29, 1879, he says: "I can hardly give you any rule about drainage, except that I do it where I expect much serous oozing, or where I am not sure that all bleeding has been stopped; and especially if, with one or other of these circumstances, the general condition is bad, or the patient very feeble."

This is a subject I have thought a great deal about, and I have lately adopted a plan to settle the question of drainage or no drainage, which I have twice carried into effect since I saw Keith operate.

Bloody serum is more dangerous in the peritoneal cavity than pure blood or pure serum, and requires drainage. If there are no adhesions to break up, the drainage tube is unnecessary. If there is ascites alone, it is unnecessary; but if we have ascites, complicating adhesions, then we may use the drainage-tube, and we are sure to have bloody serum evacuated by it. If we have adhesions alone, without ascites, and if we feel sure that every bleeding point has been secured, then we may, with the utmost confidence, close the external wound without the drainage-tube.

But if we are in doubt on this point, I think the following plan will remove the doubt. As before stated, Spencer Wells and Keith, and all their followers, place a large, flat sponge between the anterior parietes and the intestines during the time they take to introduce the sutures. In Keith's last operation, he passed sixteen sutures in six minutes. Any one else would have taken ten minutes to do the same work. During these from six to ten minutes, if the sponge that has been laid within the peritoneal cavity is found, on removal, to be perfectly dry, then we may be sure that there is no bleeding to be apprehended from the parts with which it has lain in contact. But if, as before explained, it contains two or three drachms of blood, then there are oozing vessels to be sought and tied.

To this I would add another diagnostic measure. Before introducing the large flat sponge over the surface of the intestines, pass a sponge, the size of a small orange, firmly held with locked forceps, down to the bottom of the Douglas's pouch, the handle of the forceps projecting from the lower angle of the incision. Then place the large sponge over the surface of the intestines, and proceed to pass the sutures, leaving the two sponges *in situ*. When the sutures are all introduced, draw half to each angle of the wound and remove the superficial sponge, as before described, and then squeeze it, and if it is dry, all is well in the region that it had just occupied. Then remove the small sponge, with the locked forceps, from the pelvic cavity, squeeze it, and if it, too, is found to be perfectly dry, then we may proceed to close the external wound without the drainage-tube. But, on the contrary, if the large superficial sponge contains blood, or bloody serum, then we must search out the source of oozing, and arrest it; and if we fail, we should resort to the drainage-tube. But, granting that the upper sponge is found to be dry, and the one from the pelvic cavity contains free blood, or bloody serum, then its source must be searched and secured; and if we fail, then we should resort to drainage, for I am convinced that Keith's plan of drying out the peritoneal cavity completely is the only one to give us perfect immunity against sepsis.

I have adopted this plan recently in two cases—that is, of placing a sponge in the pelvic cavity simultaneously with that on the surface of the intestines. In the first case, there was ascites, complicating most extensive adhesions between the tumor, parietes, omentum, and intestines. The upper sponge was dry; the one in the pelvic cavity contained two or three drachms, not of pure serum, but of bloody serum; and here I used the drainage-tube, and felt afterward that I had done the best thing possible for my patient; for during the first twenty-four hours after operation, there were discharged sixteen ounces of bloody serum, half of it being blood, and all of it full of bacteria. On the next day there was nearly as much more. It gradually decreased till the fifth day, when it ceased entirely, and the drainage-tube was removed. My patient recovered rapidly, the pulse never going over ninety, nor the temperature over one hundred. In the second case, there was no ascites, but there were parietal adhesions; and the adhesions between the tumor and the omentum, and between the tumor and colon and mesocolon, were very firm and very extensive, and the dissection of the tumor from the intestine was exceedingly tedious and difficult. In this case, I was in doubt whether to use the drainage-tube or not; but using the sponges as before described, the one superficially and the other deep in the pelvis, and then removing them after the sutures had all been introduced, I found that the first one was perfectly dry, while the other contained a small quantity of pure blood. As I could not find the source of bleeding, I introduced a drainage-tube. From two to three ounces of pure arterial blood passed out through this tube every day for a whole week. It did not contain bacteria; still I believe it was safer that this pure blood (twelve or fourteen ounces in all), should pass from the peritoneal cavity, than remain there, become coagulated, and take its chances of absorption. Such a case is rare, and the bleeding occurred most probably from some little arteriole on the surface of the intestine, which had been stripped of its peritoneal covering for a distance of five or six inches by two. The practice was justified by the result, as the patient recovered perfectly.

Let me hope that others may find the plan here suggested as useful in determining this most difficult question of drainage or no drainage, as it has been to me in the two cases already described. The lesson that I had gathered from witnessing

Keith's operation is—never to close the external wound till we have secured every bleeding vessel, every oozing point, and made sure that the peritoneum is perfectly clean and dry. It was long thought that the treatment of the pedicle had much to do with the success of the operation. But I think now that it matters very little what we do with the pedicle, whether we use the clamp, the ligature, or the cautery, provided we take every care against the exudation of blood or bloody serum into the peritoneal cavity after the closure of the external wound.

Spencer Wells was the great apostle of the clamp; he did more to popularize its use in the profession than any other man; he honestly believed that the extraperitoneal method of securing the pedicle was the best and safest when practicable. As late as June and July, 1878, in his lectures before the "Royal College of Surgeons," he brought forward statistics from his immense experience to prove that the best treatment of the pedicle was by the clamp, and the worst by the ligature; as the mortality attending the latter far exceeded that of the former. But Listerism has killed the clamp, and even Spencer Wells uses it no longer; or so rarely as to make its use quite exceptional. He uses the intraperitoneal ligature, cutting it off close, and leaving the pedicle within the peritoneal cavity. His pupils, Bantock and Thornton, who succeeded him in "The Samaritan Hospital," in December, 1877, adopted the antiseptic method then, and with it the intraperitoneal ligature, never having used a clamp since that time. Thus we see the two greatest ovariotomists living, Spencer Wells (with his lieutenants, Bantock and Thornton) and Thomas Keith, both treating the pedicle by the intraperitoneal method—the one by the ligature and the other by the cautery, which settles forever the question of the clamp.—*College and Clinical Record.*

